# Autonomic testing of women with interstitial cystitis/bladder pain syndrome

**DOI:** 10.1007/s10286-014-0243-0

**Published:** 2014-04-30

**Authors:** Gisela Chelimsky, N. Patrick McCabe, Jeffrey Janata, Robert Elston, Lu Zhang, Sarah Ialacci, Thomas Chelimsky

**Affiliations:** 1 Medical College of Wisconsin, 8701 W Watertown Plank Road, Milwaukee, WI 53226 USA; 2 Case Western Reserve University, 10900 Euclid Avenue, Cleveland, OH 44106 USA; 3 Department of Neurology, Medical College of Wisconsin, 9200 W. Wisconsin Ave., Milwaukee, WI 53226 USA

**Keywords:** Interstitial cystitis/bladder pain syndrome, Pelvic pain, Autonomic nervous system

## Abstract

**Purpose:**

Interstitial cystitis/bladder pain syndrome (IC/BPS) is characterized by urinary urgency, frequency, nocturia, pain worse as the bladder fills and improved after emptying. These features might suggest abnormal autonomic bladder control mechanisms. We compared the structural integrity of the autonomic nervous system (ANS) in IC/BPS and control subjects.

**Methods:**

IRB-approved study at University Hospitals Case Medical Center, Cleveland, OH to evaluate the structural integrity of the ANS in adult females. Testing included cardiovascular response to deep breathing, Valsalva maneuver, 30 min head up tilt, and sudomotor test.

**Results:**

Differences in ANS integrity for IC/BPS subjects and controls were determined by modified Composite Autonomic Severity Score (CASS) that includes sudomotor, adrenergic and cardiovascular indices. Baseline heart rate (HR) and HRs from each of three 10 min upright segments of a tilt test were compared and trend analyses performed using *t* tests. Healthy and IC/BPS subjects were demographically similar. The two groups did not differ in modified-CASS scores but elevated average peak heart rate was evident during baseline (supine; *p* = 0.057) for IC/BPS subjects prior to a tilt test. Difference at baseline was maintained at each interval during the tilt, with nearly identical slopes across intervals. The preliminary nature of this report denotes a small sample size and important differences may not be detected.

**Conclusions:**

The findings show no structural ANS abnormalities in IC/BPS subjects. Higher baseline HR supports the concept of functional rather than structural change in the ANS, such as abnormality of sympathetic/parasympathetic balance that will require further evaluation.

## Introduction

Interstitial cystitis/bladder pain syndrome (IC/BPS) classically comprises urinary urgency, frequency, nocturia, and chronic pain in the pelvis worse as the bladder fills and better once empty. Such clinical features might suggest disordered bladder control from a generalized autonomic nervous system (ANS) abnormality. This concept might explain the IC/BPS known comorbid conditions [5] such as irritable bowel syndrome, migraine headache and fibromyalgia, also associated with ANS function changes [2, 6, 10, 13]. We hypothesized that IC/BPS belongs to a larger family of (often co-morbid) disorders that share common autonomic and sensory abnormalities. This study aimed to assess the structural integrity of the ANS in subjects with IC/BPS compared to healthy age matched controls. Based on abnormalities reported in co-morbid disorders such as fibromyalgia and migraine, we expected to find changes in responses to sudomotor and tilt table testing, with preserved cardiovascular responses to deep breathing and to the Valsalva maneuver. In addition, given the changes in heart rate variability found by others in chronic pain states, we thought it important to evaluate the structure of the ANS as a basis for drawing any conclusions regarding its structure.

## Participants and methods

### Participants

This prospective IRB-approved study (University Hospitals Case Medical Center, Cleveland, OH) conducted in accordance with the ethical standards laid down in the 1964 Declaration of Helsinki and its later amendments evaluated the structural integrity of the ANS of adult females with diagnosis of IC/BPS and matched female healthy controls, as a portion of the larger ICEPAC (Interstitial Cystitis—Elucidation of Psychophysiologic and Autonomic Characteristics) study (ClinicalTrials.gov Identifier: NCT01616992). Data presented herein are from women enrolled in ICEPAC between 02/2011 and 02/2012 recruited through University Hospitals Case Medical Center (Cleveland, OH), and Case Western Reserve University (Cleveland, OH), Summa Health System (Akron, OH), and ResearchMatch.org. All subjects gave their informed consent prior to inclusion in the study. Study enrollment continues through June of 2014 with a goal of 38 healthy and 76 IC/BPS participants. Other ICEPAC subject groups are not presented here.

### Inclusion/exclusion criteria

Subjects with IC/BPS required ≥6 months of symptoms with pain clearly linked to bladder fullness, and exclusion criteria aligned with the IC/BPS-NIDDK criteria [1]. Healthy controls consisted of matched females, screened to exclude the presence of both IC/BPS and any manifestation (in the last 5 years) of any disorder commonly co-morbid with IC/BPS, including fibromyalgia by history or exam, chronic fatigue syndrome or unexplained fatigue for >6 months, chronic pain disorder of any type, complex regional pain syndrome, cyclic vomiting syndrome, irritable bowel syndrome, functional abdominal pain, functional dyspepsia, migraine headache, postural tachycardia syndrome, Raynaud’s syndrome, reflex syncope, temporo-mandibular joint disorder, post-traumatic stress disorder, panic disorder, periodic limb movements in sleep, and multiple chemical sensitivity. Exclusion criteria for all subjects (healthy and IC/BPS) included pregnancy, breastfeeding, hematuria or infection on urinalysis, three urinary tract infections within the previous 12 months, pelvic or bladder neoplasm or infection, unstable medical or psychiatric illness (such as untreated depression, psychosis, etc.), central or peripheral nervous system disorder (Diabetic Neuropathy—regardless of A1c level, Parkinson’s disease, Alzheimer’s, MS, stroke, etc.), inability to stop autonomically active or pro-kinetic (gastrointestinal motility modifying) agents prior to testing, current consumption of >10 alcoholic beverages per week, or any major surgical intervention in the last 90 days.

### Autonomic testing

All subjects underwent ANS testing described in detail elsewhere [3] including the cardiovascular responses to deep breathing (DB, primarily testing cardiac parasympathetic integrity), the Valsalva maneuver (VM, testing cardiac sympathetic, parasympathetic and vasomotor sympathetic integrity), tilt table test (testing cardiac and vasomotor sympathetic integrity) and quantitative sudomotor axon reflex test (QSART, testing post-ganglionic sympathetic cholinergic integrity). The tilt table test was performed at 70° for 30 min. The VM (using 15 s and 40 mmHg) and DB were performed 3 and 2 times, respectively, in each subject.

### Evaluation and statistical analyses

To quantify the results of testing, we utilized a modified Composite Autonomic Severity Score (CASS) [11] (Table 1), with sudomotor, adrenergic and cardiovascular heart rate indices. Some degree of anxiety may artificially inflate initial baseline pressures and heart rates, producing a false impression of an orthostatic drop. The tilt table test baseline was therefore taken from the last 3 min of the 5–10 min segment after return to the supine position, except if tilt table test was terminated due to a near-syncopal episode, when a strong vagal surge may produce a non-typical post-tilt baseline, and the baseline was taken from the final 3 min of the first 10 min supine period prior to upright tilt. The upright portion of the tilt was divided in three 10-min segments with the mean of the three highest heart rates taken as the peak for each segment. Activity and symptom severity were evaluated every minute during the tilt table test, and outliers accompanying fidgeting and talking were excluded. Subjects unable to complete the first two intervals of the tilt table test (e.g. due to a syncopal episode) were excluded from HR analysis.

**Table 1:** Modified Composite Autonomic Severity Score (CASS)

Sudomotor index	Single QSART site reduced or distal sweat volume <1/3 of proximal value
	Single QSART site <50 % of lower limit
	Two or more QSART sites <50 % of limit
Adrenergic index	Phase II_E_ reduction <40 >25 mmHg MBP, or reduced phase II_L_, or pulse pressure reduction to ≤50 % of baseline
	Increased PRT time (4–5 s)
	Phase II_L_ absent or increased PRT (6–9 s)
	Absent phase IV
	Absent phase II_L_ and IV and increased PRT ≥10 s
	The prior one plus orthostatic hypotension defined as SBP reduction ≥30 mmHg; MBP ≥20 mmHg
Cardio vascular HR index	HR_DB_ or VR reduced but >50 % of minimum
	HR_DB_ or VR reduced but <50 % of minimum
	HR_DB_ and VR reduced but <50 % of minimum

Each subsection allows for 1 score point (i.e. a total of 3 for sudomotor, 4 for adrenergic and 3 for cardiovascular HR index)

*QSART* quantitative sudomotor axon reflex, *MBP* mean blood pressure, *PRT* pressure recovery time, *SBP* systolic blood pressure, *HR*_*DB*_ heart rate response to deep breathing, *VR* valsalva ratio

Data Analysis asked several questions: (1) Do IC/BPS and healthy control HR differ at baseline or when upright? (2) Is there an upright trend? (3) If a trend is found, is it linear or quadratic and is there a between group difference in that trend? Each of these questions was answered by calculating a linear contrast of the observed average peak HR for each of the subjects in the sample and performing a *t* test. Since the average peak HR values and log-transformed values produced similar results, only the average peak HR values are shown. Age, BMI and modified CASS score between groups were also compared by *t* test.

## Results

This preliminary report includes 15 healthy women [mean age 36.3 years ± 16.1 (SD); mean BMI 26.7 ± 8.6] and 14 women with IC/BPS (mean age 36.5 years ± 16.1; mean BMI 29.0 ± 7.7). There was no significant difference in age (*p* = 0.98) or BMI (*p* = 0.46) between healthy and IC/BPS subjects. The analysis included two subjects with IC/BPS that were unable to stop medications (one was on diphenhydramine and the other on amitriptyline). The subject on amitriptyline had a modified CASS score of 3, with 2 points due to decreased sweat in the foot and 1 point for mild adrenergic dysfunction during the Valsalva maneuver, with no syncope and HR increased during the tilt of <10 bpm while upright. The subject on diphenhydramine had a modified CASS of 3 due to significant decrease in sweat output in the forearm and foot, with normal adrenergic and cardiovascular HR index, and HR increased by 33 bpm while upright prior to a pre-syncopal episode. As the results from these two subjects did not differ significantly from the other subjects, they were included for analysis. No significant difference was observed between women with IC/BPS and healthy controls for any ANS integrity test as indicated by total modified CASS scores (Table 2). Three subjects with syncopal episodes in the first 20 min were excluded from the tilt table test analysis.

**Table 2:** Summary of autonomic testing findings

	IC/BPS	Healthy	*p*
Number of subjects	14	15	
Subjects with syncope	2	1	
Subjects with orthostatic hypotension	0	0	
Sudomotor index	1.8 ± 1.3	1.4 ± 1.0	>0.05
Adrenergic index	0.6 ± 0.5	0.5 ± 0.5	>0.05
CV HR index	0.2 ± 0.4	0.1 ± 0.4	>0.05
Modified CASS	2.4 ± 1.4	2.0 ± 1.1	>0.05

Values are mean ± SD

### Cardiovascular response

Cardiac response to deep breathing was abnormal in three IC/BPS and in one healthy control. One healthy subject and one IC/BPS had a mildly abnormal Valsalva HR ratio. The cardiovascular HR index and the adrenergic index were similar in both groups (Table 2).

### Tilt table test

**Fig. 1:**
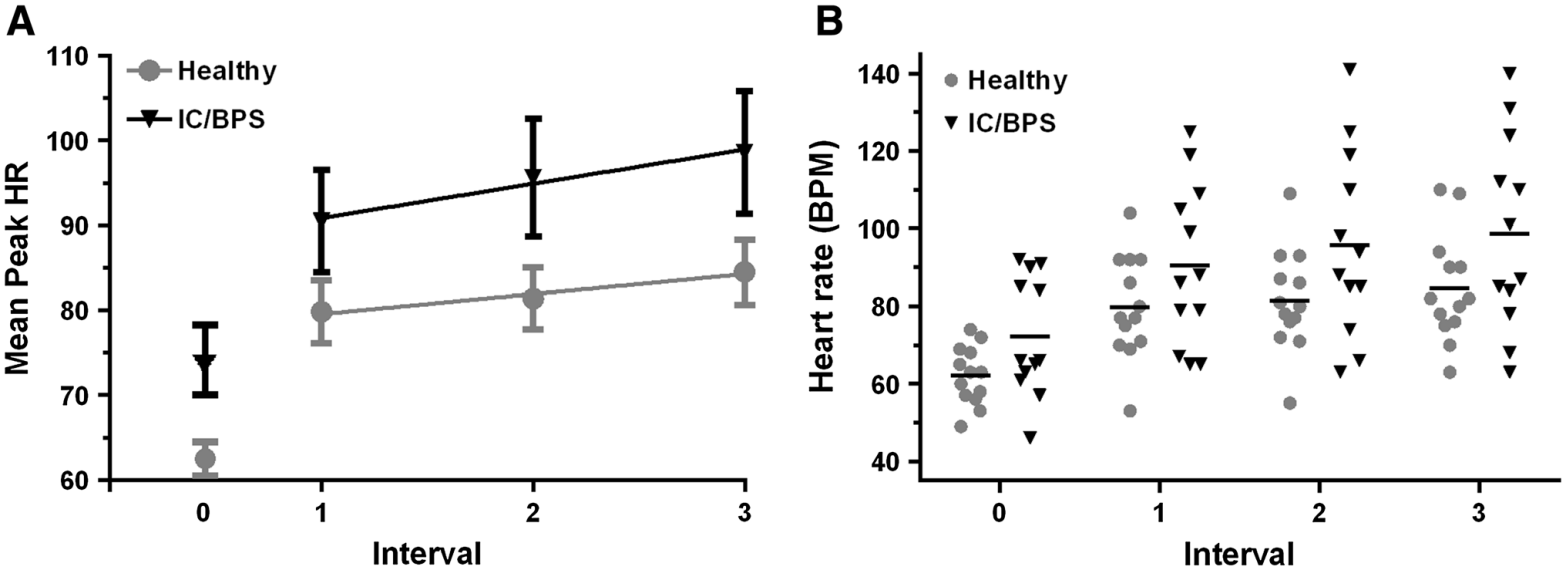
Mean peak heart rate data at baseline (interval 0) and during the first, second, and third 10-min blocks (intervals 1, 2, and 3, respectively) of the upright tilt. **a** Slopes of post-baseline trends for healthy controls and subjects with IC/BPS. Data are mean peak heart rate ± SE. **b** Raw mean peak HR data points for each group at their respective intervals

All subjects underwent a tilt table test. Two IC/BPS subjects failed to complete interval 3; one fainted 1 min into interval 3 and one complained of discomfort 4 min into interval 3 and the tilt was discontinued. Two subjects in the healthy group and 3 in the IC/BPS had pre-syncope. Orthostatic hypotension did not occur. A difference in baseline HR (supine) between the groups approached significance (*p* = 0.057) and there is a significant linear trend after baseline, with no significant non-linear trend or trend by group interaction (Table 3; Fig. 1). Log transformation produced similar results with a slightly lesser fit (not shown). The *t* tests are not independent but the correlation between contrasts for baseline and slope were not significant.

**Table 3:** Summarized data from the tilt table test

	IC/BPS	Healthy	Estimate	*p*	95 % CI
Number of subjects	12	13			
Baseline					
Peak HR	72.17 ± 15.44	62.08 ± 7.48			
Group difference^a^			10.09	0.057	(−0.352, 20.53)
Post baseline					
Peak HR interval 1	90.50 ± 20.83	79.85 ± 13.28		0.148	
Peak HR interval 2	95.67 ± 24.08	81.38 ± 13.04		0.086	
Peak HR interval 3	98.58 ± 25.02	85.54 ± 13.88		0.104	
Group difference^a^			12.99	0.106	(−3.050, 29.038)
Linear slope (pooled)^b^			3.16	<0.0001	(1.876, 4.445)
Linear slope by group interaction (non-pooled)			1.70	0.186	(−0.887, 4.278)
Quadratic curvature (pooled)^b^			−0.12	0.867	(−1.584, 1.344)
Quadratic curvature by group interaction (non-pooled)			−1.93	0.181	(−4.833, 0.968)

Peak HR data presented as mean ± SD

^a^IC/BPS minus healthy control

^b^Data were pooled (healthy control and IC/BPS) as there was no difference between the fitted lines for each group. This created a model for all data post baseline

### Quantitative sudomotor axon reflex test (Qsart)

Subjects in the healthy control and in the IC/BPS groups showed mild abnormalities of sudomotor function, with comparable modified sudomotor CASS scores (Table 2).

## Discussion

To our knowledge, this is the first study to assess ANS integrity in patients with IC/BPS. While these data are preliminary, the aim of this manuscript was to evaluate the data accumulated to date and generate hypotheses that could be tested in the complete dataset. The ICEPAC study was designed to test the hypothesis that ANS structure may be abnormal in IC/BPS. Our data to date suggest that the null hypothesis prevails, i.e. that the structure of the ANS, at least as it innervates cardiovascular and sudomotor end-organs, is normal as evaluated by standard clinical autonomic testing.

To date, several studies have assessed various aspects of the ANS in a variety of painful disorders, for example heart rate variability in fibromyalgia [15] or irritable bowel syndrome [2] or heart rate and blood pressure change to bladder filling in IC/BPS [16]. However, none of these studies had undertaken the critical preliminary step of determining whether the structure of the ANS is intact. Without this information, one could not determine whether findings of abnormal ANS activity might be due to a structural change in the ANS, or to a functional abnormality associated with the disorder being studied. The current findings indicate that ANS structure in these patients does not differ from that of healthy matched females at least in IC/BPS, suggesting that observed ANS differences more likely relate to functional, not structural abnormalities. The observed increase in the baseline heart rate, as well as a trend for increased heart rate after 10 min upright, support a tentative conclusion of an abnormality in ANS responsiveness and function, rather than any structural change in the autonomic fibers or connections themselves. This hypothesis will be further explored in separate studies focused on ANS function, using heart rate variability analysis.

It is important to note that our healthy control group, despite being highly selected by history (previous 5 years) and detailed examination to confirm the absence of any signs or symptoms suggestive of a chronic pain, autonomic or neurologic disorder, did demonstrate some mild abnormalities. Such findings challenge our understanding of “normal” [4]. Moreover, these “abnormal” values may have obscured differences between the groups. Alternatively, the absence of a difference may simply represent a type II error—a larger cohort would demonstrate a difference. The subjects with IC/BPS that could not stop medication may have augmented abnormalities associated with the tilt test and autonomic testing due to anticholinergic properties of diphenhydramine and amitriptyline. However, these medications should have increased, not decreased, the difference between the groups [7, 9].

Studies of the ANS in patients with IC/BPS are scarce. One recent study found that hydrodistention of the urinary bladder induced a much greater heart rate, systolic and diastolic blood pressure rise in subjects with and without cystoscopic findings of ulcers and/or submucosal petechial hemorrhages [16]. The authors mentioned several possible interpretations, including (1) increased (although not reported) pain sensation in patients with ulcers; (2) different stages of the same disease or (3) two different diseases. Subjects with IC/BPS also have been shown to have increased baseline heart rate at rest when compared to controls, as well as some increase in the resting diastolic, but not the systolic, blood pressure [12]. We have found similar results, with higher baseline heart rate in the IC/BPS patients. However, in none of these studies had the structure of the ANS also been evaluated. The findings from our study lend credence to the idea that one can reliably examine ANS function in these patients. The increase in heart rate may be related to several factors: (1) amount of physical activity subjects are performing; (2) a functional rather than a structural change in the ANS such as an abnormality of sympathetic/parasympathetic balance suggested by our finding and that of others [12, 16], leading to a higher baseline heart rate; (3) higher anxiety level. The findings in this hypothesis generating study will allow further exploration of these possibilities.

This is not the first study to report a change in autonomic function in patients with a chronic pain disorder. A relative skew toward sympathetic influence also occurs in other functional pain disorders such as irritable bowel syndrome, fibromyalgia and chronic fatigue [2, 8, 14]. To date, no study has examined the meaning of these findings in the context of pain generating mechanisms to understand whether increased heart rate, for example, reflects a contributor or a consequence of a chronic pain syndrome. A prospective study will clarify the role of the ANS in chronic pelvic pain, and its relationship to psychological changes that occur frequently in these disorders. Future directions might include an assessment of heart rate variability at rest and after a physiologic or psychological stress or to extend the findings reported by Lutgendorf et al. [12].

In conclusion, although this study was performed in a relatively small number of subjects (*n* = 29), which could lead to a type II error (not finding a difference when one exists) it nonetheless constitutes the largest study of the ANS in IC/BPS to date. These findings require confirmation with a larger cohort, but suggest that ANS structure is not different in subjects with IC/BPS when compared to highly screened healthy subjects. Particular note should be made that this detailed screening of normal subjects required healthy subjects to have no disorders of any kind remotely known to harbor an association with any autonomic abnormality. Nonetheless, occasional mild abnormalities were present.
